# Migraine and periodic limb movement disorders in sleep in children: a preliminary case–control study

**DOI:** 10.1186/1129-2377-14-57

**Published:** 2013-07-01

**Authors:** Maria Esposito, Pasquale Parisi, Silvia Miano, Marco Carotenuto

**Affiliations:** 1 Department of Mental Health, Physical and Preventive Medicine, Second University of Naples, Via Sergio Pansini 5 PAD XI, Naples 80131, Italy; 2 Child Neurology, Chair of Pediatrics, NESMOS Department, Faculty of Medicine & Psychology, Sapienza University, Via di Grottarossa, 1035-1039, Rome 00189, Italy

**Keywords:** Polysomnography, Periodic limb movements, PLMd, PLMs, Children, Migraine without aura, MoA

## Abstract

**Background:**

The relationships between sleep and headaches are complex and manifold. About the variety of phenomena that can disrupt the sleep macrostructure and can impact its restorative function, the periodic limb movements disorder (PLMd) can be considered as the most powerful.

No studies are known about the role of PLMd in the pathophysiology of migraine in children.

Aim of study is to assess the prevalence of PLMd and migraine and their relationship with disability and pain intensity in a pediatric sample, referred for migraine without aura by pediatricians.

**Methods:**

After a preliminary sleep habits screening with the Sleep Disturbances Scale for Children, 34 migraine subjects affected by migraine without aura (20 M, 14 F) (mean age 9.08; SD ± 2.28) and 51 volunteers healthy children (28 M, 23 F) (mean age 9.37; SD ± 1.81) accepted to underwent overnight PSG recordings in the Sleep Laboratory of the Clinic of Child and Adolescent Neuropsychiatry, in order to define the macrostructural sleep characteristics and the prevalence of PLMd. Subsequently, the migraineurs sample was studied in order to define the relationship between disability, pain intensity, therapeutical responsiveness and the presence of PLMd.

**Results:**

In the migraineurs children group, the individuals with PLM pathological index (PLMI ≥ 5) represent the 26.47% of sample and present higher frequency (p < 0.001), intensity (p < 0.001), duration (p = 0.006) and life impairment as scored in the PedMIDAS (p < 0.001) of headache and lower efficacy of prophylactic (p = 0.001) and acute (p = 0.006) pharmacological treatment than MoA children without PLM pathological index.

**Conclusions:**

This preliminary study indicates the potential value of the determination of the PLMd signs, and the importance of the PSG evaluation in children affected by migraine, particularly when the clinical and pharmacological management tend to fail in the attacks control.

## Background

The relationships between sleep and headaches are complex and manifold [1,2], with suggestions for an unique pathogenic process [3]. Among the causes of disturbed sleep in subjects affected by primary headache, the sleep apnea syndrome and the restless legs syndrome (RLS) were considered yet in 1990 by Sahota and Dexter [4].

In particular, RLS is a sensorimotor disorder characterized by an irresistible urge to move the limbs predominantly in the evening or at night, usually accompanied by a peculiar discomfort in the lower extremities often alluded to as a “creepy” or “crawly” feeling, with insomnia and daytime fatigue as a consequence [5,6]. The nocturnal polysomnography (PSG) may show the association of periodic limb movement disorder (PLMd) during the night.

According to the International Classification of Sleep Disorders - 2nd Edition (ICSD-2) criteria, Periodic Limb Movement Disorder (PLMd) is caused by periodic episodes of repetitive, highly stereotyped, limb movements that occur during sleep, with a frequency >15/h (in children >5/h), associated with a clinical sleep disturbance or a complaint of daytime fatigue [7].

Specifically, a leg movement is classified as PLM if it is part of a periodic sequence of leg movements which involve the rhythmic extension of the big toe and dorsiflexion of the ankle, occasionally accompanied by knee and hip flexion. By definition, periodic limb movements are not the result of generalized neurological disorders, which are typically apparent during wakefulness as well as during sleep [8], and defined as four or more consecutive leg movements with a duration of 0.5-10 s, with an EMG increase of >8 μV above the resting baseline, and a minimum interval of 5 s and a maximum of 90 s between two consecutive leg movements [9,10].

In general, about the variety of phenomena that can disrupt the sleep macrostructure and can impact its restorative function [9,11], the PLMd could be considered one as the most powerful.

Although the effects of PLMd have been well studied in adults, there have been limited and scarce reports in developmental age, associated with transient arousals and sleep fragmentation, which could lead to changes in daytime neurocognitive and behavioral patterns [12].

In children, the presence of PLMd may be frequently associated with low serum iron and with a tendency toward low serum ferritin levels [13].

Moreover, more conditions such as obstructive sleep apnea syndrome (OSAS), autism, ADHD, Williams syndrome, Tourette syndrome, narcolepsy, and medications like selective serotonin reuptake inhibitors, lithium and tricyclic antidepressants can be considered as risk factors for PLMd [14]. Since the description in adults in the 1980’s, their prevalence in children and adolescents is still unclear. Reported prevalence rates of the PLMd a frequency at least of 5/hr vary between 1.2% to 10% of children not referred specifically for PLMd or restless legs syndrome (RLS) [15,16], while the prevalence in children with migraine is largely unknown. Chervin and Hedger [17] reported that restlessness of the legs, growing pains in bed, insomnia, and morning headache can be considered as moderately predictive value for the identification of paediatric PLMd.

To our knowledge, there are no data about the relationship between PLMd and migraine in developmental age, and the putative common mechanisms underlying the two conditions.

Therefore, the aim of the present study is to assess the prevalence of PLMd and migraine and their relationship with disability and pain intensity in a pediatric sample of children affected by migraine without aura.

## Methods

### Study population

187 children affected by migraine without aura (MoA) (74 F, 113 M) aged 5–17 years, (mean 9.92 ± 2.86) were consecutively referred to the Center for Childhood Headache of Child and Adolescent Neuropsychiatry Clinic at the Second University of Naples, from September 2011 to December 2012. They were all referred from primary care paediatricians.

The diagnosis of MoA was made according to the International Classification of Headache Disorders. 2nd Edition (ICHD-2) [18].

All mother’s of starting population subjects filled out a questionnaire in order to assess the sleep habits (Sleep Disturbances Scale for Children, SDSC) of their children and compared with a control group composed by 766 typical developing children (342 F, 424 M; mean age 10.05 ± 2.13) comparable for age (p = 0.494) and sex distribution (Chi-square =1.374; p = 0.241), recruited in the Campania Region schools.

Therefore, from original population of 187 migraine children the subjects with SBD referred signs, EEGs abnormalities or epileptic discharges, neuro-anatomical alterations (assessed by RMN and/or TC evaluation) or psychiatric illness (depression, behavioural problems and ADHD) or mental retardation (IQ ≤70) or subjects under treatment with anticonvulsant or psychoactive drugs were excluded.

Finally, only 34 migraine subjects affected by MoA (20 M, 14 F) (mean age 9.08; SD ± 2.28) and 51 volunteers typical developing children (28 M, 23 F) (mean age 9.37; SD ± 1.81) accepted to underwent overnight PSG recordings.

All the subjects were recruited from the same urban area, were all Caucasian origin, and had middle socioeconomic status.

All evaluations were performed after informed parental consent was obtained for all the children enrolled, according to the World Medical Association [19].

The study was approved by the Departmental Ethics Committee at the Second University of Naples.

### Biochemical evaluation

In order to exclude the presence of alteration in iron metabolism (low serum iron and low serum ferritin levels), the blood samples for hemoglobin, and iron status evaluations were drawn in the morning (at 08:00 h) after overnight fasting. Hemoglobin was measured in whole blood using an automated Coulter counter, and cut-off points used to define anemia were based on the 5th percentiles for the reference groups [20].

In particular, the iron status has been assessed by serum iron, transferrin, and ferritin concentrations. Transferrin saturation has been calculated.

Particularly for serum iron, an iron ferrozine complex method was used with sensitivity of 5 μg/dl; serum transferring was measured using a turbidimetric method with sensitivity of 70 mg/dl. Serum ferritin was measured by immunometric assay. Transferrin saturation was calculated by finding the molar ratio of serum iron and twice the serum transferring (because each transferrin molecule can bind two atoms of iron) using the formula:

$$\begin{array}{c} \mathrm{transferrin}\;\mathrm{saturation}=\left[\mathrm{serum}\;\mathrm{iron}\;\left(\mathrm{micrograms}\;\mathrm{per}\;\mathrm{deciliter}\right)/\right]\\ \;\mathrm{transferrin}\left(\mathrm{milligrams}\;\mathrm{per}\;\mathrm{deciliter}\right)\\ \;\times 71.2. \end{array}$$

### Anthropometric evaluation

In order to exclude subjects with overweight or obesity, weight and height were measured and BMI was calculated. Standard deviations scores for BMI were calculated by using the LMS method [21].

### Sleep habits assessment

To evaluate sleep habits and disturbances, all mother’s of starting population subjects filled out the Sleep Disturbances Scale for Children (SDSC) questionnaire [22]. This is a standardized questionnaire for the assessment of pediatric sleep problems consisting of 26 items grouped into 6 subscales: DIMS (Disorders in Initiating and Maintaining Sleep), SBD (Sleep Breathing Disorders), DA (Disorders of Arousal), SWTD (Sleep-Wake Transition Disorders), DOES (Disorders Of Excessive Somnolence), SHY (Nocturnal Hyperhydrosis). The SDSC questionnaire is widely used in school-aged children both in the original [22,23] or as modified version [24].

The results were compared with those obtained by SDSC questionnaires of a control group composed by 766 children (342 F, 424 M; mean age 10.05 ± 2.13) matched for age (p = 0.494) and sex distribution (Chi-square =1.374; p = 0.241), recruited in the Campania region schools.

The subjects of both groups were recruited from the same urban area, were all Caucasian origin, and had middle socioeconomic status.

### Polysomnographic sleep recordings

34 migraine subjects affected by MoA (20 M, 14 F) (mean age 9.08; SD ± 2.28) and 51 volunteers healthy children (28 M, 23 F) (mean age 9.37; SD ± 1.81) accepted to underwent overnight PSG recordings in the Sleep Laboratory of the Clinic of Child and Adolescent Neuropsychiatry, after one adaptation night, in order to avoid the “first-night effect”. The groups that underwent PSG were matched for age (p = 0.516), and gender distribution (Chi-square = 0.018; p = 0.893).

As previously reported in other polysomnographic studies [25-28], electroencephalographic (EEG) recordings and electrode placement were performed according to the 10–20 system [29] and the polysomnographic montage included at least 19 EEG channels (Fp2, Fp1, F3, F4, F7, F8, C3, C4, T3, T4, P3, P4, T5, T6, O1, O2, Fz, Cz, Pz) referenced to the contralateral mastoid, left and right electro-oculogram, chin electromyogram, left and right tibialis electromyogram, electrocardiogram (one derivation), nasal cannula, thorax and abdominal effort, peripheral oxygen saturation, pulse, and position sensors.

The recordings were carried out using a Brain Quick Micromed System 98 recording machine, and signals were sampled at 256 Hz and stored on a hard disk for further analysis. EEG signals were digitally band-pass filtered at 0.1–120 Hz, with 12-bit A/D precision. Sleep signals were sampled at 200 Hz or 256 Hz and stored on a hard disk in European data format for further analysis. EEG signals were first acquired with a wide band analog filter (0.001–70 Hz) and then digitally band-pass filtered at 0.1–50 Hz. All recordings started at the subjects’ usual bedtime and continued until spontaneous morning awakening.

### Sleep stage scoring

Sleep was subdivided into 30-second epochs, and sleep stages were scored using standard criteria [30].

The following conventional sleep parameters were evaluated:

•Time in bed

•Sleep period time: time from sleep onset to end of sleep

•Total sleep time: time from sleep onset to the end of the final sleep epoch minus time awake

•Sleep latency: time from lights out to sleep onset, defined as the first of two consecutive epochs of stage 1 sleep or one epoch of any other stage, in minutes

•REM latency: time from sleep onset to the first REM sleep epoch

•Number of stage shifts/hour

•Number of awakenings/hour

•Sleep efficiency: percentage ratio between total sleep time and time in bed (total sleep time/time in bed * 100)

•Percentage of sleep period time spent in wakefulness after sleep onset, i.e., time spent awake between sleep onset and end of sleep

•Percentage of sleep period time spent in sleep stages 1 (S1%) and 2 (S2%), slow-wave sleep (SWS%), and REM sleep (REM%)

All variables were analyzed by Hypnolab 1.2 sleep software analysis (SWS Soft, Troina, Italy). All recordings were visually scored by one of the investigators (MC), and the sleep parameters derived were tabulated for statistical analysis.

In order to exclude the presence of sleep-related breathing disorders, nocturnal respiratory parameters (i.e., central, obstructive, and mixed apnea events) were counted using standard criteria [31].

The apnea-hypopnea index was defined as the number of apneas and hypopneas per hour of total sleep time; an obstructive apnea index ≥1 was selected as the cutoff for normality [32,33].

Standard criteria were used to identify episodes of periodic limb movements. The frequency of leg movements was represented as the periodic leg movement index (number/hour of total sleep time). Episodes of periodic limb movements were defined as leg movements with an amplitude increase of 8 μV above the baseline value, a duration of 0.5–10 seconds, a period length between two consecutive movements of 5–90 seconds, and a minimum of four consecutive movements [9]. A periodic leg movement index ≥ 5 was considered abnormal.

### Migraine evaluation

In the MoA group, in order to compare the headache characteristics between children with PLMd and children without PLMd (no-PLMd), we take in account the MoA frequency and the duration of headache attacks per month, the pain intensity (VAS index), the PedMIDAS score, and the subjective response to the pharmacological treatment such as acute treatment (i.e.: Paracetamol efficacy) and prophylactic treatment (Preventive therapy efficacy; i.e.: Flunarizine) obtained by the clinical interview.

The visual analogue scale (VAS) was used to assess the level of pain, by placing a mark on an horizontal line 10-cm long at an appropriate distance between the two endpoints (no pain signed as happy smiley and most intense pain imaginable signed as hopeless smiley).

The PedMIDAS is a sensitive, six-question interview that can be easily administered by both parent and child to assess the impact of recurrent headaches on a child’s life. This tool has been shown to be reliable and valid for assessing disability in children and adolescents and was found to be easy to complete and use within an active clinical setting. It can provide the impact of migraines on a child’s day-to-day activity and overall quality of life. The instrument also provides a useful tool to assess treatment outcomes and compare responses to individual therapies [34].

#### *Statistical analysis*

In order to compare the biochemical, anthropometric characteristics of both populations a t-Test and the Chi-square test, when appropriate, were performed.

To evaluate the differences in the sleep items pathological score of the SDSC in the original populations, a cut-off of at least 3 episodes per week was considered, according to the validation criteria [22]. Therefore, the Chi-Square test was used to analyse the results.

In order to select from the original sample (MoA and normal), a representative group for PSG recordings the sample size was calculated with the on line software http://www.dssresearch.com/toolkit/sscalc/size_a2.asp with an Alpha Error Level at 5% and Beta Error Level at 50%.

In the group who underwent PSG study (34 MoA vs. 51 normal children), the comparisons between sleep architecture parameters were conducted using the nonparametric Mann–Whitney U test for independent data sets [35].

In order to evaluate the PLMd influence on headache characteristics, the MoA sample was divided in two groups accordingly the periodic limb movement index (PLMI) ≥5/h [9]. Then, the t-Test and, when appropriate, the Chi-square test were performed to compare MoA characteristics (i.e. frequency, intensity, duration of attacks, life impairment and treatment efficacy) of two subgroups (MoA children with PLMI > 5/h and children with MoA with PLMI < 5/h).

In order to analyze the relationship among PLMI with frequency, duration, intensity and disability of MoA children, the Pearson’s correlation test was computed.

For all statistical analysis, p values <0.05 were considered significant.

## Results

Table 1 shows the comparison on the 26 SDSC items based on the “pathological score cut-off” (≥3/week) of both samples. MoA children show an higher quote of difficulty to falling asleep (Sleep latency ≥30 min p < 0.001, Difficulty getting to sleep at night p < 0.001, Anxiety when falling asleep p <0.001, Hypnic jerks p value = 0.009) and NREM parasomnias signs (Sleepwalking; p < 0.001 and Sleep talking; p =0.002) and sleep related movement disorders (Teeth grinding; p <0.001) in SDSC sub-items than control group.

**Table 1:** Sleep habits in migraine without aura and control children

**SDSC items**	**MoA (n = 187) (%)**	**Control (n = 766) (%)**	**Chi-square**	**p<**
**1. Sleep less than 8 h**	13.90	16.32	0.49	NS
**2. Sleep latency >30 min**	18.72	6.53	26.01	0.0000
**3. Reluctant to go to bed**	43.32	36.95	2.32	NS
**4. Difficulty getting to sleep at night**	27.27	16.45	10.94	0.0000
**5. Anxiety when falling asleep**	32.09	7.70	79.56	0.0000
**6. Hypnic jerks**	20.86	13.05	6.73	0.0090
**7. Rhythmic movements while falling asleep**	12.30	8.62	1.99	NS
**8. Vivid dream-like scenes while falling asleep**	13.90	5.09	17.01	0.0000
**9. Falling asleep sweating**	24.60	10.18	26.34	0.0000
**10. More than two awkenings per night**	17.11	12.40	2.49	NS
**11. Difficulty to fall asleep after awakenings**	17.11	12.66	2.18	NS
**12. Nocturnal hyperkinesias**	47.06	37.99	4.79	0.029
**13. Sleep breathing difficulties**	12.59	12.40	0.007	NS
**14. Sleep apnea**	4.27	4.18	0.02	NS
**15. Snoring**	17.11	17.23	0.005	NS
**16. Night sweating**	13.36	13.19	0.003	NS
**17. Sleepwalking**	14.97	6.66	12.60	0.0000
**18. Sleep talking**	28.88	18.15	10.06	0.0020
**19. Teeth grinding**	16.58	6.79	16.91	0.0000
**20. Sleep terrors**	9.09	5.09	3.65	NS
**21. Nightmares**	11.76	12.01	0.001	NS
**22. Difficulty in waking up in the morning**	33.69	32.38	0.07	NS
**23. Feeling tired awakening in the morning**	35.83	27.94	4.13	0.0420
**24. Sleep paralysis**	8.02	7.57	0.00	NS
**25. Daytime somnolence**	13.37	9.40	2.18	NS
**26. Sleep attacks**	4.81	3.13	0.82	NS

The Table 1 shows the percentage of pathological score for all the 26 items of Sleep Disturbance Scale for Children, according with the validation criteria (>3/week).

According to the sample size calculation, the size of both groups who underwent PSG (34 migraine and 51 normal children) was representative of starting population.

Table 2 shows the comparison between biochemical and anthropometric characteristics of both PSG study population.

**Table 2:** Demographic, anthropometric and biochemical evaluation in MoA and normal comparisons, who underwent the polysomnographic study

	**MoA (n = 34)**	**Control (n = 51)**	**p**
**Age**	10.67 ± 2.6	10.92 ± 2.37	0.653
**Sex ratio (M/F)**	20/14	28/23	0.893
**z-BMI (z score Body Mass Index)**	0.31 ± 0.43	0.21 ± 0.37	0.256
**Hemoglobin (g/dL)**	13.04 ± 0.97	13.1 ± 1.05	0.791
**Serum iron, (μg/dL)**	80.30 ± 17.21	79.95 ± 18.04	0.929
**Serum ferritin, (μg/L)**	34.5 ± 16.2	31.97 ± 15.1	0.464
**Transferrin saturation (%)**	23.51 ± 5.14	22.76 ± 6.32	0.571

Table 2 shows the comparison between demographic and hematologic characteristics of both PSG study population.

In order to verify the matching about sex ratio (M/F) the Chi-square test was performed; all other characteristics was evaluated applying t-Test.

Table 3 shows the statistical comparison between the sleep architecture parameters of MoA children and control group. MoA children present a reduction of TIB (p < 0.001), SPT (p < 0.001), TST (p < 0.001) with an higher quote of awakenings per hour (p = 0.008) and a higher PLM index (p < 0.001) than controls.

**Table 3:** Sleep macrostructural, respiratory parameters and PLM nocturnal evaluation

	**Migraine (N = 34)**		**Control (N = 51)**		**Mann–Whitney**	
	**Mean**	**Std.Dev.**	**Mean**	**Std.Dev.**	**U**	**p**
**TIB-min**	467.3235	46.46502	595.1961	86.57985	126.00	0.000
**SPT-min**	445.1765	47.29980	562.4412	80.85330	147.00	0.000
**TST-min**	416.3529	59.19837	534.3824	75.29167	135.00	0.000
**SOL-min**	14.6324	11.61524	22.7843	21.93109	643.5	NS
**FRL-min**	142.2206	53.99385	125.2941	50.83268	706.00	NS
**SS-h**	9.2824	2.52976	8.7686	3.44078	74.5	NS
**AWN-h**	3.8971	2.59364	2.1784	1.79881	527.5	0.008
**SE %**	88.8441	7.41261	90.0216	5.47640	845.5	NS
**WASO-spt (%)**	6.7147	7.16139	4.8471	4.08545	779.5	NS
**S1-spt (%)**	1.7882	2.07259	2.8000	3.47338	621.00	NS
**S2-spt (%)**	38.9588	9.10265	43.6451	24.84550	797.00	NS
**SWS-spt (%)**	34.7735	10.46907	30.9490	9.97830	698.5	NS
**REM-spt (%)**	17.7382	6.68839	21.1961	5.43463	620.5	NS
**AHI**	0.9512	0.35743	0.8463	0.29067	675.00	NS
**ODI**	0.3821	0.26759	0.3837	0.32744	813.5	NS
**PLMI**	18.3194	24.850	2.5333	1.11187	65.5	0.000

Table 3 shows the mean differences in sleep macrostructure, respiratory (AHI, ODI) and PLM index (PLMI) between the migraine and control group, calculated with t-Test analysis.

*TIB* time in bed, *SPT* sleep period time, *TST* total sleep time, *SOL* sleep onset latency, *FRL* first REM sleep latency, *SS* stage shifts, *AWN* awakenings, *SE* sleep efficiency, *WASO* wake time after sleep onset, *S1* sleep stage 1, *S2* sleep stage 2, *SWS* slow-wave sleep, *REM* rapid eye movement sleep percentage.

*AHI* apnea/hypopnea index (normal value≤1/h), *ODI* oxygen desaturation index (normal value≤ 1/h), *PLMI* periodic limb movements index (normal value≤ 5%). *NS* not significant.

There were not differences in sleep stage percentages and in respiratory indexes, such as apnea/hypopnea index (AHI) and oxygen desaturation index (ODI) between the two groups.

In the MoA group, the children with PLMI ≥ 5 represent the 26.47% of sample and present higher frequency (p < 0.001), intensity (p < 0.001), duration (p = 0.006) and life impairment of headache as scored in the PedMIDAS (p < 0.001), than those with PLMI < 5/h. They had also a lower efficacy of prophylactic (p = 0.001) and acute (p = 0.006) pharmacological treatment (Table 4).

**Table 4:** Comparison about pain characteristics, disability and treatment efficacy between MoA children with p values <0.05 was considered significant

	**PLMI > 5/h (9)**	**PLMI < 5/h (25)**	**p**
**Frequency attacks/month**	15.667 ± 2.236	6.840 ± 2.838	0.000
**VAS**	8.444 ± 1.014	5.200 ± 1.848	0.000
**Duration**	8.778 ± 2.108	5.320 ± 3.262	0.024
**PedMIDAS score**	63.556 ± 5.27	24.88 ± 10.41	0.000
**Paracetamol efficacy**	1/8	18/7	0.012
**Preventive therapy efficacy**	2/7	22/3	0.002

p values <0.05 were considered significant.

Moreover, the Pearson’s analysis shows a positive correlation between PLMI and frequency (*r* = .5925; p < 0.001), intensity (*r* = .4922; p = 0.003), duration (*r* = .3968; p = 0.02) and disability due to migraine (*r* = .6931; p < 0.001).

## Discussion

The main findings of our study can be summarised in the assessing of PLMd prevalence in children affected by MoA, and in the identification as potential causative role of PLMd in disability and pain intensity in children affected by migraine without aura.

In general, the inter-relationship between sleep and headache seems to interest also the sleep length: an excess or lack of sleep or a bad quality or inadequate duration of sleep could cause headache [2,4].

In fact, the short sleepers tend to exhibit significantly more frequent headaches than long sleepers and were also more likely to experience morning headaches at awakening [36].

Moreover, previous studies described the differences in sleep habits such as a higher quote of DIMS, SBD, DA, SWTD and DES categories [37], and in the sleep macrostructure such as a reduction in total sleep time (TST), rapid eye movement (REM) and slow-wave sleep (SWS), and an increased sleep latency (SOL) [38] between headache children respect of normal children, as confirmed by our findings.

In particular, our findings tend to confirm the reported differences respect of typical developing comparisons such as a higher sleep latency, higher rate in difficulty getting to sleep at night, anxiety when falling asleep, hypnic jerks, NREM parasomnias signs and sleep related movement disorders in SDSC subitems than control group. Moreover, accordingly with Vendrame study results [38], our MoA children present a reduction in TIB (p < 0.001), SPT (p < 0.001), and TST (p < 0.001) with a higher quote of awakenings per hour (p = 0.008) and PLM index (p < 0.001) than controls.

Alternatively, the PLMd have been reported with a variable prevalence in developmental age, after the first description by Pichietti and Walters [39]. Genetic factors, altered iron metabolism and dopaminergic dysfunction have been thought to be involved in the pathophysiology of PLMd.

Accordingly, in our sample all these disturbances and alterations in iron metabolism were excluded.

Moreover, the present study, first tried to assess the prevalence of PLMd according to the PSG diagnosis in children with migraine that represents the 26.47% of our migraine sample.

Anyway, the lack of normative controls for prevalence of “any PLMS” in the general population is also limiting, as we cannot confidently infer how the prevalence of “any PLMS” in children with migraine compares to the prevalence in the general pediatric population.

On the other hand, migraine may be considered a painful disabling condition, particularly in childhood, and is often accompanied by severe impairments, including low quality emotional functioning, absenteeism from school, and poor academic performance, as well as poor cognitive functioning [40,41], motor coordination, [42] sleep habits [37,38,43-49] and high maternal stress [50,51].

Otherwise, there is no consensus about the MoA origin, pathophysiology and long-term course, particularly in the pediatric population [52], even if also several brain neurotransmitter systems have been suggested to be involved in migraine, such as glutamate, noradrenaline and for neuromodulators and dopamine [53].

In this light, our data seem to suggest that PLMd could influence the migraine clinical presentation, increasing its severity, frequency and all disabling aspects, such as the treatment efficacy also.

On the other hand, a recent report pinpointed a correlation between the pain threshold in adults affected by migraine and the sleep pressure, suggesting that migraineurs on the average tend to suffer from a relative sleep deprivation and need more sleep than healthy controls [54].

## Conclusions

We should take into account the main limitation of this study represented by the fact that our subjects came from a small group from a specific region of Southern Italy.

Notwithstanding these limitations, this could be considered the first report about the potential value of the determination of the PLMd signs, and the importance of the PSG evaluation in children affected by migraine, particularly when the clinical and pharmacological management tend to fail in the attacks control.

## Competing interests

All authors declare that they have no competing interests.

## Authors’ contributions

ME concept and design and drafting the article. PP design, analysis and interpretation of data. SM editing of the manuscript. MC design, drafting and final approval of the version to be published. All authors read and approved the final manuscript.
